# Organisational learning from the public health response to the COVID-19 pandemic: findings from a qualitative interview study

**DOI:** 10.3389/fpubh.2024.1411346

**Published:** 2024-08-07

**Authors:** Felicity Southworth, Daljinder Chalmers, Gabriel Reedy, Richard Amlôt, Elena Skryabina

**Affiliations:** ^1^Behavioural Science and Insights Unit, Science Group, UK Health Security Agency (UKHSA), London, United Kingdom; ^2^Centre for Education, Faculty of Life Sciences and Medicine, King's College London, London, United Kingdom

**Keywords:** Organisational learning, learning from COVID-19, COVID-19 lessons, public health learning, qualitative phenomenological study, COVID-19, public health, major incidence

## Abstract

System learning from major incidents is essential for enhancing preparedness for responding to future adverse events. Sharing learning not only stimulates further improvements, preventing the repetition of mistakes, but may also promote collaboration and the adoption of evidenced-based best practises. As part of a qualitative interview study designed to explore lessons learned, this paper describes the experiences and perspectives of 30 staff from the public health agency responsible for the national COVID-19 response in the United Kingdom. The focus of the interviews was on enabling factors and practises that worked well, as well as those that were more challenging, and which, if addressed, could improve responses to future infectious disease incidents. The interviews elicited valuable insights across various thematic areas that could inform emergency preparedness activities for future infectious disease outbreaks. The outcomes of this study, while integral for the UK agency responsible for public health, extend beyond organisational boundaries and contribute to a broader spectrum of activities aimed at facilitating global learning from the COVID-19 response.

## Introduction

In the realm of emergency preparedness, dissemination of learning is an integral part of knowledge management practises. It is widely assumed that learning from the experience of previous incidents can help to improve practise and minimise avoidable negative impacts in future emergencies (1–3). Given that the relative rarity of major emergencies provides few opportunities to learn from direct experience, it is particularly important to ensure that learning from real life major incidents is documented and disseminated to others (4, 5). Sharing limitations and practises that did or did not work well can stimulate further improvements, which may in turn prevent duplicating previous mistakes; likewise, sharing successful practises promotes global collaboration and the adoption of best methods for successful outcomes (2, 6–8). It is also widely acknowledged that true organisational learning from emergencies is not easily accomplished: similar recurring problems are often reported across multiple incidents and timespans (2, 4, 9, 10).

After being first identified and reported in December 2019, the novel SARS-CoV-2 virus spread globally, and the disease COVID-19 was declared a pandemic by the WHO on 11 March 2020. Many countries were not prepared to deal with a highly infectious respiratory pathogen, including those with health systems largely regarded as robust (11). The pandemic caused massive public health, economic and social disruptions across the globe, thus highlighting the importance of investing in pandemic preparedness.

According to the World Health Organisation (WHO) COVID-19 dashboard [WHO coronavirus (COVID-19) Dashboard] globally, there have been over 767 million confirmed cases of COVID-19 infection, resulting in nearly 7 million deaths. Worldwide, over 13 billion vaccine doses have been administered. In the United Kingdom (UK), from 3 January 2020, there have been over 24 million confirmed cases of COVID-19, resulting in over 227,000 deaths, as reported to WHO (12). Over 151 million vaccine doses have been administered to the UK’s nearly 70 million residents (12).

During the height of the pandemic, like most health systems, the United Kingdom’s National Health Service (NHS) was under extreme pressure: staff where exhausted, wards were overflowing, and waiting times for routine and emergency care rose by over 10 times, with most procedures being cancelled or postponed due to the systemwide focus on pandemic-related care (13). Patients reported waiting more than 12 h to be seen in Accident and Emergency (A&E) departments, the highest since records began.

As a Category 1 responder, Public Health England (PHE), the governmental agency responsible for public health preparedness and response, had legal responsibility to respond to emergencies under the (14, 15). As such, the agency was one of the key partners in the UK pandemic preparedness programme, and played a central role in the response to the COVID-19 pandemic.

Both the WHO and European Centre for Disease Prevention and Control (ECDC) have encouraged the sharing of learning and experiences from the COVID-19 response (11, 16, 17). An emerging literature base has published learning outcomes from intra- and after-action reviews (IARs/AARs) of COVID-19 responses from a variety of nations (18–20) and from localised settings including hospitals, refugee camps and cruise ships [e.g., (8, 21, 22)]. Gathering the experiences of the public health response to COVID-19 from a range of perspectives and contexts helps identify common challenges and factors that enabled successful responses, which, in turn, may help to strengthen global emergency preparedness and health response in the future.

The primary aim of this study was to understand processes that could help facilitate learning from Public Health England’s (PHE) response in a major incident (MI). Two major objectives were set up to achieve this aim: first, to identify learning from PHE’s COVID-19 response, and secondly, to explore how to facilitate the implementation of learning from the MI response in a public health organisation. The first of these two objectives is reported here: this study explores the experiences of staff from inside a UK national public health agency (PHE) who were involved in the COVID-19 response, including their perspectives on enabling actors and practises that worked well (and thus should be maintained and enhanced), as well as challenges in the response and areas from which to learn for future responses.

### Public Health England’s (PHE) role in the COVID-19 response

Public Health England (PHE), an executive agency of the United Kingdom’s Department of Health and Social Care (DHSC), was established on 1 April 2013 to provide leadership for health protection and improvement, including emergency preparedness and response. The formation of PHE came as a result of the National Health Service (NHS) reorganisation in England as outlined in the Health and Social Care Act (23). PHE absorbed the role of the Health Protection Agency (HPA), the National Treatment Agency for Substance Misuse and several other agencies (23). The agency was explicitly responsible for coordination of the response to public health emergencies, including an “integrated surveillance system” and “investigation and management of outbreaks of infectious diseases” (24).

During the COVID-19 outbreak, PHE played a crucial role in the UK’s response to the pandemic. Key activities included: surveillance and monitoring the spread of the virus, including infection rates, hospitalisation and death rates; providing advice and guidance to healthcare professionals, policymakers and public on infection prevention, control, social distancing, testing and vaccination; setting up and managing testing and contact tracing facilities and the NHS Test and Trace System; working with local health teams to manage outbreaks in various settings, including care homes, schools and workplaces; and communicating vital information to the public about the virus and health protection measures. PHE was responsible for publishing weekly COVID-19 epidemiology surveillance summaries, which combined virology and mortality data from community, primary and secondary care to support national and regional planning in response to the pandemic. From April 2020, PHE collated daily reporting of the number of deaths in England where a positive COVID-19 test had been recorded (25).

In August 2020, the UK government announced the reorganisation of public health protection in England, leading to the abolition of PHE in March 2021. In October 2021, PHE’s health protection functions were formally transferred into the United Kingdom Health Security Agency (UKHSA), while its health improvement functions were transferred to a number of other government agencies, including the Office for Health Improvement and Disparities (DHSEC), NHS England, and NHS Digital (26). The decision to replace PHE was reportedly due to the organisation’s performance early in the pandemic, and challenges implementing measures to test, track, and trace the disease (27). This led initially to the establishment of NHS Test and Trace and the Joint Biosecurity Centre, which ran contemporaneously with PHE, until amalgamated into the UK Health Security Agency in October 2021.

## Method

This phenomenological study design consisted of semi-structured interviews with a purposive sample from among Public Health England (PHE) staff involved in the COVID-19 response.

Ethical approval for the study was provided by PHE Research Ethics and Governance Group (PHE REGG R&D 427). Informed consent was obtained from all participants prior to the interview. Participants were informed of confidentiality, anonymity, and their right to withdraw from the study.

### Participants

A purposive sampling method, with snowball sampling, was used to recruit study participants. This included PHE staff who were (a) involved in the COVID-19 response, and (b) in a position to participate in, or influence the implementation of, organisational learning identified from the response. Individual invitations to take part in the research included information about the purpose and procedure of the interviews. Individuals who were willing to participate then liaised with the research team to answer any further questions about the study and to schedule a suitable time for the interview.

Initially, 28 individuals (14 senior leads, 14 tactical leads) were identified based on their expertise and invited to take part in the study. A second wave of recruitment involved approaching additional key informants recommended by these initial participants because of their relevant experience and potential to contribute valuable insights. Out of 51 potential participants invited, only 30 were able to take part in the interviews. This number of participants satisfied the theoretical requirements for reaching data saturation in the phenomenological study (28). No further recruitment was conducted beyond this point.

### Interviews

Semi-structured conversational interviews were conducted based on a topical guide and sought to explore the experiences and perspectives of participants. This approach is appropriate for a phenomenological study (29), which seeks to encourage participants to articulate their experiences openly and to avoid introducing too many prior assumptions or biases from the research team.

The interview topic guide (see Supplementary material 1) was developed by the research team and piloted with two agency colleagues to ensure that it was comprehensive, relevant, easy to follow, and clear. The guide included some potential questions to gather participants’ background data, and some proposed open-ended questions separated into two main parts. In the first part, participants were invited to discuss their perceptions of the agency’s COVID-19 response, including what they thought had and had not gone well, what they believed had impeded the most effective response, and what could have been improved or done differently. In the second part, participants were invited to discuss how they believed organisational learning from a major response could be facilitated. The topical guide for this second part was based on Kitson’s model of organisational learning (30), which considers factors such as evidence gathering, actioning and facilitation of implementing lessons, and organisational context factors as central features of organisational learning (please see Supplementary material; Supplementary material 3 for additional information on Kitson’s framework). At the beginning of the interview, participants were asked to describe their professional background, their usual role in PHE, and their role in the COVID-19 response. All interviewees were directly involved in the COVID-19 response through their roles, with five holding various strategic roles and 17 in tactical roles. Eight colleagues had Emergency Preparedness Resilience and Response (EPRR) positions as their primary roles. The average length of their experience within their roles was 4 years, ranging between 0.5 and 15 years. All participants were in a position to contribute to the identification and implementation of learning from the COVID-19 pandemic.

All participants were provided with a Participant Information Sheet that explained the purpose of the study and the intention of the interview. They were also invited to reach out to the research team if they had any questions or concerns. Written consent was obtained from all participants before the interviews were conducted. No additional contact with interviewees took place before the interview.

Interviews, scheduled for approximately an hour, were conducted remotely using a videoconferencing meeting platform (Microsoft Teams) between July and November 2021, by a trained and experienced interviewer (DC), who was employed by the agency for this purpose as an independent researcher who had not been involved in the pandemic response. Thirteen participants completed the interview within the one-hour initial session, whilst 16 participants completed the interview across two hourly sessions. One interview was only partially completed, as the participant was unable to schedule a second session to complete it, however, the partial data was still included in the analysis. The mean interview length was 82 min (range 23–130 min). Interviews were audio recorded and transcribed verbatim. Due to technical problems, two interviews were only partially recorded and transcribed; these were included for analysis.

### Data analysis

The transcribed interview data were thematically analysed using the following approach: (1) becoming familiar with the data; (2) generating initial codes; (3) searching for themes; (4) reviewing themes and (5) defining and naming themes (31). Analysis was conducted in the context of a sensitizing framework consisting of two pre-determined major themes: (i) enabling factors contributing to the organisational COVID-19 response, and (ii) challenges and barriers to an effective response. Within these two major categories, themes and sub-themes were generated inductively using open coding, allowing for as many codes as necessary to characterize the data.

To generate initial codes, three members of the research team were each randomly assigned three interview transcripts. Each researcher familiarised themselves with their assigned interviews and generated a list of codes to support the data analysis. The researchers then discussed identified codes together and generated an initial framework of themes and codes.

Full coding of interview data was then conducted using NVIVO V.11 software by FS, who is trained in qualitative data analysis. Coding was conducted inductively, using the explicit or surface meaning of the data, and allowing for codes to be adapted or added throughout the process as necessary to characterize the data. Once initial coding was complete, themes were explored and discussed with researcher ES. Codes were then reviewed and refined by FS to simplify the coding framework and identify emerging themes. Two researches (FS & ES) worked collaboratively to group the codes into descriptive themes and agree on appropriate theme names that accurately reflected the content of each theme. FS identified relevant interview quotes to support each theme.

Coding reliability and validity were checked by an independent experienced researcher who independently applied the generated codes to three randomly selected interview transcripts. Excellent coding agreement was achieved for all codes across all three transcripts, with a mean Kappa of 0.997 (range 0.75–1.00), indicating a high level of agreement and consistency.

Major themes identified from the data are described below, and supported by relevant quotes from participants.

## Results

The sensitizing framework for data analysis consisted of two main concepts: (i) enabling factors and (ii) challenges and barriers. Major themes within each of these concepts are described below in order of their prominence in the data and supported by participants’ quotes. (Table 1, Supplementary material, Supplementary material 2 provides a summary of the major themes, along with the number of sources and references for each theme).

### Enabling factors

Many themes within the ‘enabling factors’ concept had parallel themes within the ‘challenges and barriers concept (see Table 1, Supplementary material; Supplementary material 2). Two key themes, however, were predominantly described as enabling factors: ‘staff commitment’ and ‘scientific and technical expertise’. These themes are described in the sections that follow. Other key themes within the ‘enabling factors’ concept (‘response co-ordination,’ ‘identifying and learning lessons,’ ‘communication and collaboration,’ ‘wellbeing and staff support,’ and ‘leadership’) are described and contrasted alongside their parallel counterparts in the ‘Challenges and Barriers’ concept.

#### Staff & organisational commitment

Many participants praised the dedication and commitment of staff involved in the response. They highlighted their willingness to work hard, and to go above and beyond what might be expected of them, including working exceptionally long hours. Participants identified a significant presence of goodwill amongst their colleagues across the organisation, and noted that colleagues were committed to doing their best. This commitment also enabled resources to be redirected to the response (e.g., releasing staff and office space, and covering ‘business as usual’ roles).

*“…it’s shown how many dedicated staff we have who are just willing to go above and beyond and work themselves tirelessly, …to support this” –* Tactical Lead

Participants highlighted that the organisation was responding under difficult circumstances – both in terms of the unprecedented scale of the pandemic, and against a background of under-resourcing, political and social criticism, and wide-ranging uncertainty. Participants gave examples of how the organisation, and individuals within it, had risen to these challenges and demands, doing the best it could with what it had available. The fact that the organisation was able to deliver the response despite these challenges was seen as a success.

*“I do believe that PHE was committed to doing the very best it could at that time, with all the challenges that we were presented with”* – Senior Lead

#### Scientific & technical expertise

Participants noted the high-quality, globally recognised scientific expertise present in the organisation. Participants highlighted particularly important scientific work conducted by the agency, including serological studies, sequencing work to identify variants of the virus, and vaccine effectiveness studies. Some also praised the organisation’s ability to provide high quality scientific and clinical advice, which informed policy and government guidance, which was valued and respected by policymakers and decision-makers. They also highlighted the contributions of data scientists and statistics experts, particularly in the development of new data sharing technologies (i.e., data dashboard) and contact tracing.

*“This has demonstrated our ability to provide the scientific advice and support which is required to inform national policy making in the pandemic. And that ability to synthesise data, produce guidelines, work with politicians has been phenomenal” –* Senior Lead

Participants attributed these scientific and technical successes to the breadth of internal skills and expertise in the organisation. In addition, the organisation’s pre-existing global scientific reputation enabled it to work with international collaborators to support its scientific work.

*“So, what I think has gone well is our ability to mine all the public health skills within PHE, whether those are data, epidemiological, negotiating and influencing, analysts, evidence, behaviour… because those skills were there, and those capacities and capabilities were there, I think we have made incredibly good use of them”* – Senior Lead

### Challenges and barriers

#### Human resource

Participants highlighted issues with staff capacity and under-resourcing in multiple functions and teams, as staff worked to provide the surge capacity needed to meet the demands of the response.

*“I think in terms of what’s impeded the most effective PHE response in this outbreak has to be—one hundred percent—capacity, and the fact that we just do not have the staff” –* Tactical Lead

*“…Years of just slashings in the public health budget meant that the capacity to do what needed to be done… it just wasn’t there”* – Senior Lead

Participants also highlighted the importance of ensuring the right mix of skills in the organisation, emphasising quality as well as quantity. Some participants noted challenges in areas where there was a scarcity of skills and expertise, particularly specialist roles such as emergency planners, incident directors, and experts in areas like infectious diseases and genomics. There is frequently a national shortage of these highly specialised staff, making training and recruitment challenging. Participants emphasised the need for long-term investment in developing these skills to ensure that qualified staff are available when needed.

*“You cannot just pluck them off a tree and say, right, we are going to put some money for this purpose now, let us go and grab all these people. They’re not there, because we have not developed them”* – Tactical Lead

Others suggested that resourcing issues could have been improved by the organisation mobilising its existing skills and capacity more effectively. Better strategic awareness of the availability of skills, along with better awareness of where skills and resources were most needed, could have improved the response.

Resourcing challenges were also exacerbated by difficulties with staff recruitment and onboarding needed to meet the increased demands of the pandemic response. Participants noted that internal organisational delays in recruitment were a major barrier to scaling up the response, along with difficulties attracting candidates to roles with short-term contracts and unfavourable terms and conditions.

*“At every stage of the response we have been working on a short-term plan […] never had time to bring people in on longer term contracts so they have got stability, so we can attract better people to the roles”* – Tactical Lead

#### Planning & preparedness

Many participants highlighted issues associated with planning, both before and during the pandemic. While preparedness plans were focused on influenza and high consequence infectious diseases (HCIDs), the specific details of the COVID-19 pandemic meant that these plans were not as applicable as they could have been. Participants identified the need for more generic, flexible pandemic plans which could be adapted to a wider range of potential pathogens, and which included the ability to respond to the unknown. Some participants suggested that more detail was needed in plans (e.g., standard operating procedures, governance arrangements, lesson reports from previous incidents), and highlighted the importance of not diverging from plans once they were set, changing strategically only when necessary.

*“We had a process in place for something which was called a high consequence infectious disease response developed as a result of Ebola, but I was concerned at the time, and I remain [concerned], thinking that that did not work as well as it should have done because it was very focused on the NHS care for a small number of highly ill people. […] and the flu pandemic clearly wasn’t fit for the purpose of following […] so I think we had a sort of fundamental gap in planning strategy”* – Senior Lead

*“We did not have a plan, other than our incident response plan […]. But a lot of the other stuff that we needed to have in place, like the community testing, these are all stuff that should be commissioned probably anyway, but have not been, historically. And so, they were having to start at a point of having very little or nothing in place”* – EPRR

*“When we came to set up cells at the beginning of the response, there was a paucity of supporting information. It was a blueprint for the structures and some top line information on what different bits the response do, and the governance structures. But there was nothing beneath that”* – Tactical Lead

Many participants also highlighted a particular gap in surge capacity planning that would be needed to meet the excess demand of the response (as discussed previously under the theme of “Human Resource”). Participants identified the need for greater reserve capacity, along with clear plans for how to effectively mobilise staff resources and urgently bring in additional capacity. They also emphasised the need for better workforce preparedness, ensuring that staff had sufficient skills and training to enable the organisation to respond effectively, including familiarisation with pandemic response plans. Some participants suggested that in the future, more staff in the organisation needed to be trained as Category 1 responders and Incident Directors.

*“We did not have staff trained, we do not have any mandatory training for Category 1 responders, despite the fact that we are all Category 1 responders, and could at any moment be asked to stand up and support any kind of response”* – Tactical Lead

Other areas for improved preparedness raised by participants included network building (i.e., developing internal and external collaborative partnerships in advance), improved infrastructure (i.e., facilities, equipment, and technology), and plans for business continuity (i.e., arrangements for ensuring essential functions are maintained). Finally, participants suggested there was a need for greater commitment to future pandemic planning and preparedness, to ensure that the organisation could be better equipped to respond to future incidents.

#### Leadership & strategic response

Participants highlighted the significance of the overall strategic approach, direction, and high-level incident coordination, especially during the early stages of a pandemic. However, they noted that there were areas for improvement. Participants reported that they saw organisational leaders engaged in reactive and operational-level work, leaving less capacity to establish and maintain a higher-level strategic view. Others highlighted the importance of defined command and control structures, with clear lines of responsibility for various aspects of the response.

*“Certainly, for some time at the beginning […] I think everybody was, sort of, running around and being very busy. But that’s my point, that you do need people to take a strategic view, and not everybody to be fully bogged down in operational issues.”* – Senior Lead

*“Allowing seniors to be freer to conduct some of that high level planning […]. I think that we needed a bit more of a structured objective and direction” – EPRR.*

Participants also highlighted the importance of transparency and accountability in decision-making, and clarity on how decisions are made and by whom, which was not as clearly present as they would have liked. Others felt that due process in decision making was important and should be followed, and that it seemed as if some individuals had undue influence on decision-making. Some participants noted that unsuitable directives resulted from instances where all relevant individuals were not involved in decision-making processes.

*“I think […] there has been from the outset a lack of clarity about how decisions were being made. And it does feel that a lot of decisions were not made, [or] were made informally”* – Senior Lead

*“… expertise as regional directors [was initially not] really used as effectively as they could be, as part of the central coordinating core to help to shape policies that would be locally acceptable and locally relevant. So many times, it felt as though things were being designed … And then we were asked to implement, and then we said we cannot, because this does not make sense, it’s not going to land well”* – Senior Lead

Some participants also highlighted how important it was that leaders were aware of organisational operational activities and available resource capabilities, so that they could support strategic decision-making. Senior leadership engagement with front-line staff, both in terms of listening to staff needs and views, and explaining requests and decisions, was also highlighted as being important.

*“I do not think even within the organisation there was sufficient understanding of what our capabilities were, what our capacity was […]. I do not think they, themselves, even knew what we could do, or we could not do”* – Senior Lead

*“So many times, when the decisions that are made, there’s no concept of the operational implementation of that. And our teams have so often been left like, floundering and going, well, we have not got, what about this question, then? We’re getting these questions. All the very predictable questions that you are gonna get with a policy change, and no one national has considered them”* – EPRR

Participants also suggested that strong leadership was needed to manage the expectations of government and ministers, particularly concerning the organisational remit and resourcing. Good representation, both in terms of political and public visibility, was seen as important to ensuring the organisation could play its role as a nationally respected voice for public health.

*“should have been the people that could turn around to the secretary of state and say, well not actually, PHE is completely under resourced for doing this […] If you want this, you are gonna need to be able to either pour some more resources into PHE or do something else about it”* – Tactical Lead

Some participants provided examples of successful leadership and praised stand-out individual leaders. These leaders were noted as being engaged with staff, having a comprehensive understanding of the response, and enabling effective strategic direction and response management.

#### Communication & collaboration

Communication and collaboration emerged as a prominent theme, reflecting challenges experienced both internally and externally, including with relevant partners and stakeholders (such as the NHS and other government departments). Participants suggested that there was a need for better connections between different functions and specialities within the organisation, to facilitate joined up working and ensure that different parts of the organisation communicated effectively with each other. Participants particularly highlighted a divide between regional and national functions, suggesting a need for better engagement with and between regions to help shape policies that were locally relevant, whilst also maintaining sufficient alignment and consistency between regions. Some participants also mentioned that different parts of the organisation did not have a shared understanding of priorities, leading to difficulties with cohesive working and suggesting a need for more effective top-down communication. Similarly, many suggested a need for stronger relationships and lines of communication with external partners, to ensure the public health voice was heard across the board, along with better shared understanding around where responsibilities for different aspects of the response lie.

*“I think that the relationship with the regions was not strong enough, in the beginning of the response. In fact, well into the response. Again, I would say late summer 2020 is when that started to be focused on and, really, people concentrated on the working between the regions and the center and that’s when that got better”* – Senior Lead

*“I think there is an issue around the internal communications. How we have communicated to our staff participating in the response. You know, what our priorities were, what we needed them to do”* – Senior Lead

*“There was very little good understanding of what, you know, what the entirety of Test and Trace were doing, and where the interactions and the interfaces were between PHE and Test and Trace. And whose responsibility was what, for certain things”* – Tactical Lead

Where successful communication was noted, it was attributed to positive relationships, whether pre-existing or built during the response. These relationships enabled open communication and better understanding between collaborating partners. Strong command and control structures, along with clear roles and responsibilities, were also seen as facilitators of effective collaborative working.

Participants also highlighted the need for better information sharing, both internally and externally, and improving access to data and information. Participants highlighted the need for improved information flows, coordinated through a centralised, easily accessible system. Whilst the dashboard was mentioned as a useful information resource, and its development was seen as an aspect of the response that had gone well, it was noted that this was not available from the start of the incident and would have helped the initial response. In addition, there was a need for targeted and efficient information dissemination, with participants noting the burden involved in compiling and reporting information. Coordinated information sharing could have reduced duplication and stopped routine information sharing when it was no longer necessary.

*“I think certainly information flows, particularly at the beginning, were really, really difficult. Information wasn’t necessarily coming through […] and also there was a lot of conflicting information, and information not necessarily making sense”* – Tactical Lead

Participants also highlighted the need for improved public communications and community engagement, which would have helped to develop public trust and ensure that guidance and recommendations are understood and accepted by the public. In addition, some participants highlighted how public-facing staff should have advance notice of any changes to guidance and should be equipped to handle queries from the public.

*“Thinking about how we were engaging with communities […] the initial response was so biomedical, and so focused on testing and tracing rather than understanding and supporting communities to be part of the response.” –* Senior Lead

#### Working conditions & welfare

Many challenges identified in this theme were due to under-resourcing (as earlier reported in the “Human Resource” theme). Many participants raised issues with staff being burdened with excessive workloads, regularly working excessively long hours. Significant concerns were raised about the impact of these pressures on wellbeing (e.g., high levels of stress, exhaustion), along with the impact on the quality of work when staff are overstretched, including the increased risks of mistakes being made or tasks being overlooked.

*“I think the intensity of what people were working on and the hours that people were working was something that was a concern”* – Tactical Lead

*“We end up going from one year to the next with senior people being desperately over-stretched. Working conditions that we would normally say would be very unhealthy and unthinkable”* – Tactical Lead

Other wellbeing challenges included the potential for traumatic exposure for some staff involved in the response (e.g., hearing about and giving advice on upsetting topics), and the impacts of the pandemic on life outside of work (e.g., relatives and friends who were shielding or seriously unwell from the virus, social isolation due to lockdowns). There were also reduced opportunities for face-to-face support due to working from home, and some staff faced challenges with working in a home environment (e.g., lack of space, childcare).

*“The way in which we were all living and working presented many challenges to people on a personal basis […]. I mean it was a very, very stressful time”* – Tactical Lead

Due to these various welfare challenges, participants highlighted the importance of robust wellbeing support for staff. Many participants acknowledged the organisation’s efforts to support the wellbeing of staff during the pandemic, particularly through encouraging a culture of peer support, and a good level of support provided by line managers and leadership. Participants also highlighted the important role of the wellbeing survey to monitor staff wellbeing, along with constructive messaging, signposting, and provision of wellbeing services. However, participants also suggested that there was a need to further improve support for staff, including both stronger wellbeing messaging and making formal welfare provisions available.

*“I think one of the things that I feel that we have not done as well as we could is the mental health support to staff. I think staff have really, really struggled”* – Tactical Lead

#### Learning lessons

Participants raised areas where they felt the organisation could have better addressed lessons that had previously been identified, either from exercises or previous major incidents. Many noted that previously identified issues around staff capacity, deployment of skills, and training (e.g., from Ebola response) did not appear to have been implemented by the organisation as lessons learned. Several participants also suggested that easier access to lessons reports from the Ebola incident could have made setting up incident response structures and processes (e.g., response cells, SOPs, governance arrangements) more efficient.

*“We should have taken on board learning from previous incident responses where we did have shortages of staff and we did have real challenges around resourcing […]. And that learning came out over and over again out of every incident response but was never really addressed” – Tactical Lead*

Many participants also thought that the organisation needed to do better at learning and implementing lessons throughout the COVID-19 response, although others believed that the organisation had learned and improved throughout the response. In particular, there was frustration that lessons were being raised and not acted upon in a timely manner, along with complaints about lessons not being shared or communicated across the organisation. Overall, participants generally felt that learning was inconsistent across the organisation, happening in some places but not others.

*“We’re gathering all of these lessons, but I do not know if we are waiting until the end with a massive list, or whether it might’ve been better to have regular small chunks that could be actioned now. And where are we getting updates? Where can we see that these have been taken forward, considered and put into action, and who’s monitoring it?”* – Tactical Lead

The establishment of the lessons identified team in the early stages of the pandemic response was seen as a positive step forward to support organisational learning from the pandemic, and the lessons identified survey, mailbox, and debriefs were viewed as useful methods of capturing learning. However, the absence of pre-existing mechanisms to enable learning right from the start of the incident was viewed as a limiting factor. Others felt that there was insufficient resourcing of lessons identified team, which limited the capacity to deliver organisational learning objectives. Some participants also suggested a need for more engagement from senior leadership in the lessons identified process and more buy-in from senior leaders to promote learning across the organisation.

*“I think if we had anticipated the need for a live lessons process before, rather than trying to build it in flight, that would’ve made a huge difference”* – Tactical Lead

*“My personal opinion is that the needs of a successful lessons identified program were not fully appreciated. So, the resourcing of the team was not adequate to deliver what you would hope to deliver, or what we were being asked to deliver”* – EPRR

#### Governance

Participants highlighted the need to improve incident-related governance structures and processes in the organisation, including: record keeping and decision logging; ensuring accuracy and consistency in information; and sign-off on guidance and communications. It was suggested there was a need for better oversight and accountability, with more clarity about who was responsible for governance: indeed, some participants suggested that governance arrangements of the response were too complex or opaque. Participants also felt that the organisation needed to improve its governance culture in general, with better commitment to good quality governance and understanding of governance issues.

*“I think probably more understanding from the beginning about good governance …, and quality within one’s own cell. But I think you can only really have that if people have a good understanding of governance and quality in their day-to-day job”* – Tactical Lead

One particularly prominent area of concern was governance relating to risk management: participants raised concerns about staff needing to keep risk registers up-to-date, even during the response, as well as concerns about consistently recording adverse incidents and near misses.

#### Remit of roles & expectations

Multiple participants reported that key stakeholders and partners did not understand the purpose and role of the organisation within the national public health system. As result, participants reported that they were sometimes asked to do things that seemed not to be within the organisation’s remit, whilst at the same time new government organisations were being established for activities which participants thought should have been within the agency’s existing remit. In addition, some participants felt there was often a mismatch between the organisation’s capacity and some expectations that stakeholders or policymakers had (e.g., testing capacity), resulting in reputational damage when expectations were not met. Participants suggested that the organisation needed to be more proactive and clear in articulating and communicating its purpose and capabilities, to ensure that its role in the response was understood by members of the public and others in government and the health system.

*“There was a point where new organisations and teams were being established to do the work that we were supposed to do. And a converse was true, that because people did not know what we did, they ascribed responsibility to PHE when it wasn’t our responsibility”* – Senior Lead

#### Incident coordination

Participants reported mixed views about incident coordination. Many participants felt that the response had been well-coordinated, and noted that the establishment of the National COVID-19 Response Centre (NCRC) and the formation of response cells played a vital role in coordinating the response. However, many other participants described challenges with response coordination. They reported that different parts of the response did not fully align with each other, resulting in lost efficiency, duplicated effort, lack of focus, and misunderstanding between functional teams. Participants suggested that improved planning and simple response structures, along with clearer command and control, would improve response efficiency and help maintain a consistent response structure throughout the incident. Where response coordination was seen as having gone well, participants emphasised the importance of having existing tried-and-tested plans and arrangements which could be implemented, whilst also highlighting the importance of flexibility and agility in adapting plans to meet unique or unexpected challenges.

*“I would have wanted us, in retrospect, to just switch, an operational switch, and go, this is a really top-level response. Every part of PHE, this is now how it works”* – Senior Lead

*“One of the key things we need to learn is that arrangements for the response to outbreaks and incidents, however complex those outbreaks and incidents are, the arrangements for leading and coordinating the response should be as simple as possible. And I think the complexity of the response led to confusion, to duplication, led to misunderstandings, and also, in itself, sucked a lot of resources”* – Senior Lead

#### UK government

Some participants felt that there was insufficient understanding across government of what was required for an effective response from a public health organisation, resulting in challenging decision making in some areas and accountability issues in other areas. These misunderstandings were seen as impeding an effective response, both for organisational operation decisions (e.g., resourcing, decision to re-arrange response structure) and for the broader pandemic response (e.g., delays to implementing lockdowns, border controls).

*“I do not think you can dissociate [it] from the relationships that the response has had to have with central government and decision-making […]. So I think that’s impeded… not so much the way PHE has done its work, but the sense of it being asked to do the right things. Or make sure that what its done is being used most effectively”* – Tactical Lead

Some participants also reported the challenges of feeling unfairly blamed or scapegoated in the pandemic response. It was also felt that organisational demands were sometimes unrealistic, because organisational capacity was not sufficient to meet expectations. This sense of unjustified blame had a negative impact on staff morale and caused reputational damage to the organisation.

#### UKHSA transition

Various participants noted that the transition from Public Health England (PHE) to United Kingdom Health Security Agency (UKHSA) was a disruptive challenge, adding to existing work pressures, because preparing for the transition generated additional work in an already resource-stretched environment. In addition, the transition generated uncertainty for staff, including concerns about job security, which further reduced staff morale and engagement, and led to concerns about staff recruitment and retention. Many participants thought the decision to transition from PHE to UKHSA during an ongoing major incident response was particularly challenging.

*“I think the transition, the improvements that we are going to get out of the transition, and there are some, I think they could have been done without winding up an organisation and starting a new one. So, I think that’s a major mistake. It’s weakened our position; it’s going to lead to a staffing crisis if we are not very careful”* – Tactical Lead

## Discussion

The purpose of this interview study was to understand experiences of staff members from a national public health agency who took part in the COVID-19 response. The intention was to identify practises that worked well, and thus should be maintained and enhanced, as well as challenges that should be addressed to improve future infectious disease incident responses. Prominent enabling factors identified included staff commitment and scientific and technical expertise. Areas where challenges were reported included human resource capacity; planning and preparedness; leadership and strategic response; communication and collaboration; working conditions and welfare; learning lessons; governance; organisational remit; incident coordination; UK government; and the transition from PHE to UK Health Security Agency during the pandemic response.

The most prominent enabling factor was the dedication and commitment of staff, which allowed the organisation to rise to the challenges presented by the pandemic. This echoes previous findings from a mass casualty terrorist incident response, which reported similar attitudes and commitment from the National Health Service (NHS) staff who participated in the response (32). Thus, dedicated and committed staff appear to be an important asset to public health organisations and are an important enabler of a successful major incident response, and must be supported and encouraged accordingly. However, significant concerns were also raised in the present study regarding excessive workloads and staff being over-stretched during the pandemic, which has a potentially negative impact on staff wellbeing and on the quality of work. A recent study by Anchour et al. (33) conducted with NHS staff found a risk of losing significant staff capacity during extreme events if staff needs are not considered. While goodwill and dedication are central to the efforts of any public health system, public health organisations should be careful not to over-rely on the dedication and goodwill of staff, and should not consider these as a substitute for adequate resourcing.

Indeed, the most prominent challenge reported in the present study was limited staff capacity, due to chronic under-resourcing and a lack of surge capacity to meet the increased resource demands of the pandemic. Previous research has identified difficulties with surge capacity as a recurrent problem in responses to infectious disease outbreaks (6), and this issue has also been reported in other published literature on lessons from the COVID-19 pandemic (18, 19). In contrast, where adequate surge capacity was available at the start of the pandemic, this has been reported as a key factor in enabling an effective response (20). Given that the scale of the COVID-19 pandemic has required a greater public health resource commitment over a more sustained period than any previous major health incident, the COVID-19 response appears to have exposed a previously under-recognised weakness in the UKs pandemic preparedness. A priority recommendation, based on this research, is to strengthen surge capacity planning in the UK for pandemic and infectious disease responses. Other authors have suggested that public health responses should be reconceived as if they were a defensive combat response, meaning that public health organisations retain significant reserve capacity during ‘peacetime’ to allow for rapid expansion during an emergency (6). Our findings support this proposal.

Allied with this, another prominent challenge reported here was gaps in planning and preparedness, indicating a need for greater investment in pandemic preparedness. Previous research has also highlighted robust planning and preparedness, including comprehensive major incident plans and training, as a key factor in determining successful incident response and coordination (2, 7, 9, 19, 22, 34). Recommended areas for improvement in pandemic planning and preparedness based on this research include surge capacity planning, workforce training, and incident coordination structures, including appropriate governance arrangements. These findings also suggest that pandemic plans need to be able to be adapted for a wider range of potential public health scenarios. Addressing these issues with planning and preparedness should help to improve several other areas where challenges were reported, including mobilization of resources, strategic response, incident coordination and internal clarity of roles and responsibilities.

Various challenges were also reported regarding communication and collaboration, including internal collaboration; engagement with external partners and stakeholders; information sharing; and public communications. Challenges with communication are a common theme in the literature on lessons learned from a variety of different types of major incidents (2, 6, 8, 9, 18, 19, 21, 32). The recurrence of this problem points to the importance of effective communication in a MI response and suggests a general need for further research on optimising communication and collaboration in emergency responses.

Previous research has also identified leadership challenges as a common cause of difficulties in major incident responses, including: the need for clear direction from leaders; the need for clarity in leadership roles; and the need for clear command systems (6, 9). Examples of successful leadership were provided by participants in the present study. However, participants reported that strategic response leadership was a challenge to an effective response. This indicates a need for further development in these areas, and for more clear command-and-control structures. It is important to identify what characteristics a strategic leader needs: Davies and Davies (35) reported that strategic leaders have the ability to strategically orient themselves and their teams, are able to translate strategy into action, are able to align employees and the organisation to the strategy, are able to determine when an effective intervention would be deemed useful, and are able to help develop these same competencies in others. Similarly, highlighting the significance of good strategic leadership within an emergency response environment, fundamental lessons identified from the work by Parker et al. (36) show that acting and responding swiftly is paramount, and that effective major incident (MI) leaders should be good communicators who are practical, adaptable, and who practise daily reflection. Whilst leadership challenges may be partly addressed by improvements in resourcing and planning, these findings also highlight the importance of leadership being transparent and accountable, engaging with staff, having robust organisational knowledge, and managing expectations.

In addition, the present findings highlighted the importance of effective governance structures and processes, along with a culture of governance to ensure that processes and procedures are followed. Previous research has also highlighted strong governance as crucial for supporting effective emergency response, and inadequate implementation of governance processes such as monitoring and auditing have been found to cause failure in incident response (8, 9, 19). There is a need for more commitment to good quality governance, along with training to ensure an adequate understanding of governance issues, particularly in risk management. Governance processes and culture should be strengthened outside of major incidents, to ensure that good governance becomes routine.

Consistent with findings from previous incidents, this study also emphasizes the importance of psychological wellbeing support for staff during major incidents (32, 37). Our findings indicate that some helpful support was available, particularly in the form of peer support, which has been previously identified as an important supporting mechanism to ease the negative impacts on staff emotional health and wellbeing, that can result from participating in a MI (37–39). Further reinforcement of psychosocial wellbeing provision for health organisation staff during Mis, including through improved access to appropriate training, resources and services, could improve the situation.

The present study reported mixed findings with regards to learning lessons from the pandemic response. Whilst there were some reports of organisational learning and improvement throughout the response, there were also challenges with implementation of lessons in a timely manner. Participants identified that inconsistent organisational learning was likely due to the pressures of the ongoing pandemic response, lessons not being shared across the organisation, and lessons identified from previous incidents and exercises having not being addressed. The establishment of a lessons identified programme from the early stages of the response was viewed as a positive step in enabling learning from the pandemic, and this initiative should be maintained and enhanced for future MIs. The translation of ‘lessons identified’ into ‘lessons learned’ is a widely recognised global problem, with recurring problems often being reported across multiple incidents. This demonstrates that learning from major incidents is not easily accomplished (2, 4, 9, 10). Furthermore, many of the challenges identified here, such as resourcing, communication, leadership, and governance, have also been identified as crucial factors for facilitating organisational learning from a MI response (4, 40–43). Based on the outcomes of this study, we suggest that addressing these challenges will not only strengthen organisational emergency preparedness to respond to future major incidents, but will also help reinforce appropriate context, structures, and mechanisms necessary for effective organisational learning.

The various challenges and enabling factors described in this study add to an emerging international literature base on learning from the COVID-19 response, by contributing the perspectives of staff from the UK’s public health agency who were central to the national response. Much of the currently published literature on learning from the COVID-19 pandemic involves reporting outcomes from intra-and after-action reviews (IARs/AARs) following the WHO methodology (8, 17–22), whereas the present study involved one-to-one interviews with staff involved in the ongoing response. By taking a different approach, this study can supplement outcomes from IARs and AARs of the COVID-19 pandemic, and aid in triangulation of findings.

### Strengths & limitations

In this phenomenological study exploring the perceptions and experiences of staff participating in a national COVID-19 response, the research team took steps to ensure that the research was conducted with a high level of rigour, especially since study aimed to explore the organisation within which they were employed. The research team also included experienced researchers from outside the organisation, for instance, and a researcher independent to the team helped add additional rigour to the coding process. The study had a relatively large sample size for qualitative research (*N* = 30), which provided sufficient data for the team to be confident in the analysis and results. The study captured the views of staff working directly on the pandemic response, and did so whilst the response was still active, hopefully reducing the risk of recall bias. The data reflected the apparent willingness of participants to provide thoughtful and critical responses, including their perspectives on their own missteps and problems in the response, showing that confidentiality and anonymity arrangements allowed staff to share their views openly and honestly.

As a phenomenological study, the present study relied on the self-reported experiences and views of staff involved in a national COVID-19 pandemic response. The study did not seek to include participants from other agencies or bodies included in the response who were external to PHE, and who may have different perspectives on the national response. The study researchers were also employees of or collaborators with PHE, and whilst steps were taken to ensure that the data collection and analysis were rigorous and thorough, and the interpretation and conclusions sound, this is a potential limitation of the study. A combination of purposive and snowball sampling was used to recruit the study participants. While this recruitment method allows for the selection of knowledgeable informants, it may potentially introduce selection bias. This study focuses on the UK national public health agency, which offers a specific organisational context. Applicability of the findings to other contexts and settings should be done thoughtfully and carefully.

### Conclusion

Our paper reports experiences by the leading UK public health agency during the response to the COVID-19 pandemic, through a rigorously designed research study. The study identified unique challenges for PHE responders, as well as highlighting those identified during previous public health responses. The latter indicates that more work is needed to apply lessons previously identified, and this is reinforced by new data from the pandemic response. In addition, this paper provides a valuable perspective from the UK, contributing to an evidence base which is currently dominated by research from North America.

National capacity and capability to respond to infectious disease outbreaks is essential for the protection of public health. The present study portrays enabling factors and challenges in the public health COVID-19 response based on the views and experiences of public health agency staff involved in the response. This study highlighted the importance of understanding staff experiences during a pandemic response, to address critical issues and facilitate a thorough post-incident analysis. Participants emphasised the importance of strengthening surge staff and planning capacity for pandemic and infectious disease responses, which will help meet the potential demands of future incidents, and the necessity of investing resources in timely learning from the response as well as the implementation of learning.

Sharing identified lessons is an essential element of learning from emergency responses and strengthening global preparedness for the future. However, given how challenging it is for organisations to truly learn lessons from emergencies, further work is required to explore how best to ensure that learning from the COVID-19 pandemic is translated into practise. In an accompanying paper (Southworth et al., Submitted) we continue to develop this dataset to identify strategies for facilitating the implementation of learning from the COVID-19 response. Learning identified and reported in this study can be used as first-hand evidence by a broader audience, including policymakers and public health practitioners, to inform global public health emergency preparedness efforts.

## Data availability statement

The original contributions presented in the study are included in the article/Supplementary material, further inquiries can be directed to the corresponding author.

## Ethics statement

The studies involving humans were approved by the Public Health England’s Research Ethics and Governance Group (PHE REGG R&D 427). The studies were conducted in accordance with the local legislation and institutional requirements. The participants provided their written informed consent to participate in this study.

## Author contributions

FS: Formal analysis, Investigation, Visualization, Writing – original draft, Writing – review & editing. DC: Data curation, Investigation, Writing – original draft, Writing – review & editing. GR: Methodology, Resources, Writing – review & editing. RA: Conceptualization, Methodology, Supervision, Writing – review & editing. ES: Conceptualization, Data curation, Investigation, Methodology, Resources, Supervision, Validation, Visualization, Writing – original draft, Writing – review & editing.
