# Supplementary material for: Organisational learning from the public health response to the COVID-19 pandemic: findings from a qualitative interview study
Source: Front Public Health. 2024 Aug 7;12:1411346. doi: 10.3389/fpubh.2024.1411346 (PMC11339791; doi:10.3389/fpubh.2024.1411346)
Supplement: Supplementary file 1 [file Data_Sheet_1.docx]

**Supplementary File**

**Supplement 1: Interview Topic Guide**

**PHE organisational learning from the COVID-19 outbreak response**

**Welcome**

1. Thank the participant for agreeing to taking part in the interview and confirm that I have received their completed consent form. Check they have read the participant information sheet and ask if they have any questions.

2. Explain the nature of the study:

“Before we start, I’d just like to remind you that your participation is voluntary, and you can stop this interview at any time without giving an explanation. All information that you give will be confidential, and any published data from this interview will be anonymous. I would also like to remind you that we are audio recording this interview with a digital recorder – once the interview has been transcribed, we will delete the recording. The interview should take no more than 45 minutes”.

3. Start digital recorder.

4. State participant number.

5. Explain the purpose of the research:

“In this research we are hoping to explore PHE learning from its response in the COVID-19 outbreak and identify factors that facilitate organisational learning from a major incident.

There are no right or wrong answers; we just want to learn about your experiences and views based on your own experience in your role. The interview follows the premise of being a ‘safe learning environment’ so we really want you to be free to say it as it is, and to be as open and honest as you can. Your individual answers will not be shared with PHE.

1. **General background**

“*I’d just like to start by getting a few background details about you*.”

What is your normal daytime role?

How long have you been in your role?

What is your role in the response? Response cell? (Directorate, length of employment, grade

How long have you been involved in the response?

1. **Learning from PHE response in the COVID-19 outbreak**

Can you tell me what do you think has gone well in this response from a PHE perspective? (a few key highlights that you have identified)

Is there anything you would consider that has not gone well in this response from a PHE perspective?

What do you think has impeded the most effective PHE response in this outbreak and why?

What could have been improved to make ongoing PHE response in the outbreak more effective?

Within your role what could have been done differently to have a more effective response from your team?

Do you think PHE fostered a culture that enabled deep learning from the response to take place?

**3). How can we facilitate real-time learning during the response? (Specific prompts for):**

1. **Evidence gathering**

What type of evidence do you think would be needed to support real-time learning during a response? How do you think such evidence should be collected (e.g., regular staff surveys (if so with whom, how often), interviews/debriefs (with whom, when, how often?)

Do you think we need a different type of evidence which can support post-incident learning? If so, how can this evidence be gathered and from whom?

1. **Facilitation of learning implementation**

How can real-time learning during an ongoing response be facilitated and what support should be in place to implement the learning?

Who should be involved in the actioning of identified lessons and how the whole process should be conducted during the response: (is it the same process for the lessons at operational level and at strategic level)?

What would be the best way to implement the identified actions: (think documentation, sharing, knowledge, reporting)

- during the response
- post-response

1. **Organisational context**

Are there any organisational factors that you think are important in facilitating real-time actioning lessons and learning implementation? If yes, what are these factors?

How would you describe the PHE culture? Do you think we are a learning organisation?

What are the three most important factors that should be in place if we want to create a learning organisational culture and promote real-time learning?

What are the factors that promote long-term organisational and system learning?

**End**

1. End of interview. Try to provide some summary of the interview.

2. Thank the participant for their time and ask if they have any questions or suggestions.

**Supplement 2:**

Table 1: Summary of major themes, along with number of sources and references for each.

| **Theme** | **Summary** | **Sources*** | **Refs**.** |
| --- | --- | --- | --- |
|  |  |  |  |
| ***Enabling Factors*** | |  |  |
| Incident Coordination | Internal coordination; coordination regional level response; coordinating national response; governance, assurance, and oversight; flexibility, agility, adapting existing plans; existing tried and tested response plans. | 19 | 31 |
| Staff & Organisational Commitment | Dedication and commitment of staff; willingness to work hard; good intentions; rising to the challenge, responding under difficult circumstances; support and goodwill from outside the response. | 17 | 30 |
| Identifying & Learning Lessons | Acting on identified lessons; learning and improving throughout the response; establishing lessons identified programme; lessons identified survey, mailbox; engagement with staff in lessons identified; debriefs. | 18 | 29 |
| Scientific & Technical Expertise | Scientific advice (e.g., informing guidance, policy etc.); skills and expertise; high quality scientific work; serological testing; sequencing, identification of variants; data and statistics; behavioural and social science; vaccine development; existing global reputation and status; scientific networks. | 18 | 26 |
| Communication & Collaboration | Internal communication and collaboration; virtual working; with regions, local authorities etc.; with external partners; synthesizing data and information. | 14 | 23 |
| Wellbeing & Staff Support | Peer support, staff supporting each other; support from line managers, leadership; wellbeing survey, monitoring wellbeing; wellbeing messaging, signposting; provision of wellbeing services; working from home improving wellbeing. | 5 | 16 |
| Leadership | Stand-out individual leaders; leadership direction, response management, strategy; engagement with staff; representing the organisation. | 8 | 13 |
| Support Services | HR services; IT services and infrastructure. | 7 | 12 |
| Initial Response | Rapid mobilization; early contact tracing, testing; early recognition. | 9 | 10 |
| ***Challenges & Barriers*** | | | |
| Human Resource | Limited capacity, under resourcing; scarcity of specific skills or expertise; mobilization of skills and existing capacity; role gaps; lack of surge capacity; recruitment; onboarding & induction. | 27 | 109 |
| Planning & Preparedness | Insufficient planning, reactive response; workforce preparedness (skills, expertise, training); surge capacity planning; unsuitable existing pandemic plans; not sticking to existing plans; preparedness to adapt to the unknown; pre-investment in pandemic preparedness; network preparedness (i.e., building relationships); infrastructure preparedness; business continuity planning. | 24 | 85 |
| Leadership & Strategic Response | Direction and strategy; incident coordination; communicating priorities; decision making; accountability and transparency; provision and distribution of resources; managing expectations; aligning goals and objectives to capacity; prioritizing resources; organisational understanding; representation and public visibility; listening to staff; diverging from existing plans and arrangements; leadership role gaps; clarity in leadership roles and responsibilities; role modelling behaviors; explaining actions and decisions. | 21 | 83 |
| Communication & Collaboration | Internal (e.g., between teams, cells, departments); external liaison (e.g., with other government departments); with regions; public communication; information sharing; guidance; access to data and information; communication style (messaging, giving advice); community engagement; cross regional collaboration. | 25 | 82 |
| Working Conditions & Welfare | Excessive workloads, overstretched staff; negative impacts of work pressures on wellbeing; provision of welfare support; inappropriate behaviour; impacts on quality of work, increased risk of mistakes; negative impacts of working from home on wellbeing (e.g., isolation); reward and recognition; traumatic exposure at work; working from home challenges; promoting wellbeing messages; impacts of pandemic on wellbeing. | 20 | 73 |
| Learning Lessons | Learning throughout the response; not acting on lessons; learning from previous incidents; senior leadership buy-in and engagement; communication of identified lessons; absence of pre-existing lessons identified mechanism; undervaluing and under-resourcing of lessons identified; joined-up lessons identified; previously identified lessons not implemented; learning from exercises. | 21 | 54 |
| Governance | Culture of governance (e.g., commitment, understanding); oversight and accountability; risk registers, risk management; tracking and monitoring; record keeping, decision logging; due process, following existing procedures and processes; information governance; sign-off on decisions, comms, guidance etc.; overly complex or unsuitable governance structures; adverse incident governance. | 18 | 52 |
| Remit of Roles & Expectations | Understanding of organisational role and purpose; clarity of roles and expectations; understanding of capacity, mismatch between expectations and capacity; understanding of responsibilities and requirements of others (e.g., other government departments). | 19 | 47 |
| Incident Coordination | Disorganised response structure: slowness to mobilize; ensuring policies and procedures were feasible to implement; provision of support functions; re-arranging response structure; command and control structures. | 18 | 37 |
| UK Government | Government response (e.g., planning, strategy); taking responsibility and accountability; focus on pleasing government; understanding in government; blaming or scapegoating; introducing additional functions; unrealistic demands. | 17 | 35 |
| UKHSA Transition | Transition management (e.g., HR, staff retention); exacerbating work pressures; queries over necessity of transition; impact on staff engagement, morale. | 13 | 30 |
| Technical Resources & Facilities | Technical and scientific facilities and infrastructure; difficulty adapting existing facilities. | 10 | 17 |
| Testing & Tracing | Lack of testing capacity; understanding where testing capacity sat; understanding limits of testing. | 9 | 15 |
| Equality, Diversity & Inclusion | Health inequalities; diversity and inclusion in the workplace. | 6 | 12 |
| Media Representation | Scapegoating or blame; representation of testing; impact on morale; reputational damage. | 8 | 10 |

*Sources are participants who were interviewed (total number of participants was 30)

** References identify frequency with which a specific topic/theme has been raised across all sources

**Supplement 3:**

**Kitson and Harvey theoretical framework**

Kitson et al (1998) framework has been used to explore factors that could affect learning implementation in the context of a public health organsiation. According to the model, successful implementation of learning occurs as a function of the interplay between three factors: the nature of the evidence to support the learning (that includes research, clinical experience, patient preferences); the mechanisms which facilitate learning implementation, and the context (i.e., environment or setting) in which learning occurs. These concepts have been extensively studied, applied and further developed (Kitson et al., 2021). These developments contributed to the Promoting Action on Research Implementation in Health Services (PARIHS) framework (Harvey & Kitson, 2020). The framework emerged from observing successful implementation of learning and innovations in healthcare settings and is based on the same three key concepts (evidence, context and facilitation).

The PARIHS framework has made a significant contribution to the field of implementation science by providing a robust theoretical foundation for studying and understanding the complex process of implementing evidence-based practice, and remains a valuable tool in the field of healthcare implementation that keeps evolving and further refined (Duan et al, 2022).

**Reference List**

Duan et al. (2022). Conceptual and relational advances of the PARISH and i-PARISH frameworks over the last decade: a critical interpretive synthesis. Implementation Science.17:78; 1-25

Harvey, G., & Kitson, A. (2020). Promoting action on research implementation in health services: the integrated-PARIHS framework. In *Handbook on implementation science* (pp. 114-143). Edward Elgar Publishing.

Kitson A, Harvey G, McCormack B. (1998). Enabling the implementation of evidence-based practice: a conceptual framework. *Qual Health Care*, 7(3):149-58.

Kitson et al. (2021). How nursing leaders promote evidence‐based practice implementation at point‐of‐care: A four‐country exploratory study." *Journal of advanced nursing* 77.5 (2021): 2447-2457
