# Supplementary material for: Organisational learning from the public health response to the COVID-19 pandemic: findings from a qualitative interview study
Source: Front Public Health. 2024 Aug 7;12:1411346. doi: 10.3389/fpubh.2024.1411346 (PMC11339791; doi:10.3389/fpubh.2024.1411346)
Supplement: Supplementary file 2 [file Table_1.DOCX]

Consolidated Criteria for reporting qualitative studies (COREQ): 32 item checklist

| No. | Item | Guide Questions/description | Page No |
| --- | --- | --- | --- |
| **Domain 1: Research Team and Reflexivity** | | | |
| Personal Characteristics | | | |
| 1. | Interviewer/facilitator | Which author/s conducted the interview or focus group? | 7 |
| 2. | Credentials | What were the researchers credentials? E.g. *PhD, MD* | 30 |
| 3. | Occupation | What was their occupation at the time of the study? | 30 |
| 4. | Gender | Was the researcher male or female? | - |
| 5. | Experience and training | What experience or training did the researcher have? | 7 |
| Relationship with Participants | | | |
| 6. | Relationship established | Was a relationship established prior to study commencement? | 7 |
| 7. | Participant knowledge of the interviewer | What did the participants know about the researcher? E.g. *personal goals, reasons for doing the research?* | 7 |
| 8. | Interviewer characteristics | What characteristics were reported about the interviewer/facilitator? *E.g. bias, assumptions, reasons and interests in the research topic* | 7 |
| **Domain 2: Study Design** | | | |
| Theoretical framework | | | |
| 9. | Methodological orientation and Theory | What methodological orientation was stated to underpin the study? E.g. *grounded theory, discourse analysis, ethnography, phenomenology, content analysis* | 6 |
| Participant selection | | | |
| 10. | Sampling | How were participants selected? E.g. *purposive, convenience, consecutive, snowball* | 6 |
| 11. | Method of approach | How were participants approached? E.g. *face-to-face, telephone, mail, email* | 6 |
| 12. | Sample size | How many participants were in the study? | 6 |
| 13. | Non-participation study | how many people refused to participate or dropped out? Reasons? | 20 people were not able to take part in the study; PHE was in the response and facing a major organisational change, this significantly affected colleagues’ availability |
| 14. | Setting of data collection | Where was the data collected? E.g. *home, clinic, workplace* | 7 |
| 15. | Presence of non-participants | Was anyone else present besides the participants and researchers? | No |
| 16. | Description of sample | What are the important characteristics of the sample? E.g. *demographic data, date* | 7 |
| Data Collection | | | |
| 17. | Interview guide | Were questions, prompts, guides provided by the authors? Was it pilot tested? | 6 |
| 18. | Repeat interviews | Were repeat interviews carried out? If yes, how many? | N/A |
| 19. | Audio/visual recording | Did the research use audio or visual recording to collect data? | Yes, |
| 20. | Field notes | Were field notes made during and/or after the interview or focus group? | Field notes were not kept in the study |
| 21. | Duration | What was the duration of the interviews or focus group? | 7 |
| 22. | Data saturation | Was data saturation discussed? | The study had a relatively large sample size for qualitative research (N=30), which provided sufficient data for the team to be confident in the analysis and results. |
| 23. | Transcripts returned | Were transcripts returned to participants for comment or correction? | Transcripts were not returned to participants for comments or corrections. The final manuscript was offered for review and comments to selected number of participants. No corrections were required. |
| **Domain 3: Analysis and Findings** | | | |
| Data Analysis | | | |
| 24. | Number of data coders | How many data coders coded the data? | One, FS, pg 8 |
| 25. | Description of the coding tree | Did authors provide a description of the coding tree? | Table 1 |
| 26. | Derivation of themes | Were themes identified in advance or derived from the data? | Inductive approach, |
| 27. | Software | What software, if applicable, was used to manage the data? | 8 |
| 28. | Participant checking | Did participants provide feedback on the findings? | As per point 23 |
| Reporting | | | |
| 29. | Quotations presented | Were participant quotations presented to illustrate the themes/findings? Was each quotation identified? E.g. *participant number* | Yes |
| 30. | Data and findings consistent | Was there consistency between the data presented and the findings? | Yes |
| 31. | Clarity of major themes | Were major themes clearly presented in the findings? | Yes |
| 32. | Clarity of minor themes | Is there a description of diverse cases or discussion of minor themes? | No |
